# Is Lever Test Superior to Lachman, Pivot Shift, Drawer Tests in Diagnosing Anterior Cruciate Ligament Injuries?

**DOI:** 10.7759/cureus.22049

**Published:** 2022-02-09

**Authors:** Deniz İpek, Sinan Zehir, Abdulrahim Dündar

**Affiliations:** 1 Department of Orthopedics and Traumatology, Hitit University, Erol Olçok Training and Research Hospital, Corum, TUR

**Keywords:** pivot, lever, lachman, anterior drawer, anterior cruciate ligament injuries

## Abstract

Introduction: The physical examination in anterior cruciate ligament (ACL) injuries is extremely important, and the Lever test is commonly utilized on ACL evaluation. However, the number and scope of studies on the Lever test is limited. In this prospective cross-sectional study, we aimed to evaluate the effectiveness of the diagnostic values of Lachman, Pivot Shift, Lever, and Anterior Drawer tests in terms of quadriceps atrophy and case phase in ACL injuries.

Methods: In this prospective study, diagnostic values of Lachman, Pivot Shift, Lever, and Anterior Drawer tests were examined on 189 patients with positive MRI results as the gold standard.

Results: Lever test positivity was significantly more frequent in the group with quadriceps atrophy preoperative and after sedation (p<0.05). Anterior Drawer test positivity was significantly more frequent in the group with positive quadriceps atrophy preoperatively, after sedation and after spinal anesthesia (p<0.05). Lever and Anterior Drawer tests were positively correlated with quadriceps atrophy preop and after sedation (p<0.05). Lever test before surgery, after sedation and after spinal anesthesia in the chronic patient group was more positive than in the acute and subacute groups (p<0.05). Lever test was positively correlated with phase preoperatively, after sedation and after spinal anesthesia (p<0.01).

Conclusion: The presence or absence of quadriceps atrophy in patients with acute, sub-acute, or chronic ACL injury has a significant effect on the predictive value of the Lever test. We think that univariate analyzes may give incorrect results when demonstrating predictive value, and it would be more correct to perform multivariate analyzes.

## Introduction

The anterior cruciate ligament (ACL) is one of the primary ligaments of the tibio-femoral joint and has an important role in the motion and stabilization of the knee [1-8]. ACL injuries are one of the important health problems affecting the quality of life of individuals, and many reasons can lie in the etiology from injuries to wrong movements in sports activities. The Lachman, Pivot Shift, Drawer, and Lever tests are important for both risk factors and early diagnosis [9-17]. Studies on predictive value are carried out for early and correct intervention in ACL injuries and to avoid unnecessary invasive procedures [18]. Although MRI is used as the gold standard, the importance of early predictive value is clear. The tests with reported predictive values that are most emphasized in the literature may be listed as Lachman, Pivot Shift, Lever, and Anterior Drawer.

Among these tests, early studies have demonstrated that the Lever test was 100% consistent in predicting ACL rupture as compared to Anterior Drawer tests [11,19], however, studies in the following years reported lower predictive values. Abruscato et al. [20] reported the sensitivity as 0.77 and the specificity as 0.90 for the Lever test in their meta-analysis, in which they examined eight clinical studies. Therefore, although they generally have high predictive values, the predictive values of the methods show some differences.

Although there are studies on the predictive values of Lachman, Pivot Shift, Lever, and Anterior Drawer tests, no studies have been found on the effect of phase (acute-subacute-chronic) and quadriceps atrophy variables on the predictivity of these tests. Therefore, the aim of this study was to evaluate the efficiency of Lachman, Pivot Shift, Lever, and Anterior Drawer tests according to quadriceps atrophy and case phase in cases of ACL rupture.

## Materials and methods

This prospective study was initiated following the approval (21.05.2020/252 approval number) of the local ethics committee. We aimed to evaluate the effectiveness of the diagnostic values of Lachman, Pivot Shift, Lever, and Anterior Drawer tests in terms of quadriceps atrophy and case phase in ACL injuries. For this purpose, 189 patients who were scheduled for surgery with a diagnosis of total ACL rupture and accepted to participate in the study were included in the study. Patients with multiple ligament injuries, patients with ACL injury in both knees, patients with partial ACL rupture, patients with previous surgery around the knee, patients with previous arthroscopic knee surgery, and patients with bleeding disorders such as hemophilia were excluded from the study.

Total rupture of the ACL was confirmed by MRI in all patients. A code number was given to the patients at the time of admission while recording demographic data, they were divided into acute, subacute, and chronic according to the time of ACL injury. Quadriceps atrophy of all patients was evaluated and recorded. Both knees of all patients were examined by two different clinicians independently and blindly. Patients were examined in the clinic, under sedation on the operating table and after spinal anesthesia. Lachman, Pivot Shift, Lever, and Anterior Drawer test results were recorded separately.

Statistical methods

In the study, frequency analysis was used to define nominal and ordinal data, and mean and standard deviation values were used to define the age. Kolmogorov Smirnov test was performed for normality distribution of age parameter. Since the distribution was normal, the independent sample T-test was used for two-group differences, and the one-way analysis of variance (ANOVA) was used for phase groups. Levene test was performed for variance homogeneity in independent sample T-test and ANOVA test. Differences between groups were made using the chi-square test and chi-square similarity ratio tests. Differences within groups were analyzed using the Wilcoxon signed-rank test. Spearman's rho correlation analysis was used in the relational screening analyzes. All analyzes were performed using SPSS 17.0 (SPSS Inc., Chicago, IL, USA) for Windows program and at 95% confidence interval.

## Results

Chronic phase was significantly more common in the quadriceps atrophy positive group (p<0.05). Lever positive at preop and after sedation were significantly more common in the quadriceps atrophy positive group (p<0.05). Anterior Drawer positive at preop, after sedation and after spinal were significantly more common in the quadriceps atrophy positive group (p<0.05) (Table 1). 

**Table 1:** Age, phase, direction, and predictive value differences between quadriceps atrophy positive and negative groups

	Quadriceps atrophy positive (n=40)	Quadriceps atrophy negative (n=146)	Total (n=186)	p
Age, mean ± SD	30.20±10.02	29.08±7.00	29.32±7.73	0.508^a^
Phase, n (%)				
Acute	-	53 (36.3)	53 (28.5)	
Subacute	16 (40.0)	82 (56.2)	98 (52.7)	0.000^b^
Chronic	24 (60.0)	11 (7.5)	35 (18.8)	
Direction, left, n (%)	22 (55.0)	92 (63.0)	114 (61.3)	0.357^b^
Lachman preop, n (%)	39 (97.5)	130 (89.0)	169 (90.9)	0.063^c^
Lachman after sedation, n (%)	39 (97.5)	134 (91.8)	173 (93.0)	0.163^c^
Lachman after spinal anestezi, n (%)	40 (100.0)	146 (100.0)	186 (100.0)	>0.05
Pivot preop, n (%)	35 (87.5)	108 (74.0)	143 (76.9)	0.072^b^
Pivot after sedation, n (%)	40 (100.0)	145 (99.3)	185 (99.5)	0.486^c^
Pivot after spinal anestezi , n (%)	40 (100.0)	146 (100.0)	186 (100.0)	>0.05
Lever preop, n (%)	38 (95.0)	104 (71.2)	142 (76.3)	0.002^b^
Lever after sedation, n (%)	38 (95.0)	103 (70.5)	141 (75.8)	0.001^b^
Lever after spinal anestezi , n (%)	38 (95.0)	123 (84.2)	161 (86.6)	0.077^b^
Anterior drawer preop, n (%)	33 (82.5)	93 (63.7)	126 (67.7)	0.024^b^
Anterior drawer after sedation, n (%)	40 (100.0)	126 (86.3)	166 (89.2)	0.001^c^
Anterior drawer after spinal anestezi , n (%)	40 (100.0)	138 (94.5)	178 (95.7)	0.046^c^

According to Spearman's rho correlation analysis performed for the relationship between the parameters with significant differences and Quadriceps atrophy, preop and after sedation Lever and Anterior Drawer parameters were positively correlated with quadriceps atrophy (p<0.05) (Table 2).

**Table 2:** Spearman’s rho correlation analysis between quadriceps atrophy, phase, preop and after sedation Lever and Anterior Drawer parameters

Quadriceps atrophy	r	p
Phase	0.525^**^	0.000
Lever preop	0.230^**^	0.002
Lever after sedation	0.235^**^	0.001
Anterior drawer preop	0.165^*^	0.024
Anterior drawer after sedation	0.182^*^	0.013
Anterior drawer after spinal anestezi	0.111	0.132

Patients without quadriceps atrophy was the least common in the chronic group with statistically significant difference (p<0.05). Lever at preop, after sedation and after spinal were more positive in chronic patient group than acute and subacute group (p<0.05) (Table 3).

**Table 3:** Age, without quadriceps atrophy, direction and predictive value differences among phase groups a. One-way analysis of variance (ANOVA) test, b. chi-square test, c. chi-square likelihood ratio, SD: standard deviation.

	Acute (n=53)	Subacute (n=98)	Chronic (n=35)	p
Age, mean ± SD	28.38±6.36	29.55±7.86	30.09±9.22	0.546^a^
Without Quadriceps atrophy, n (%)	53 (100.0)	82 (83.7)	11 (31.4)	0.000^b^
Direction, left, n (%)	34 (64.2)	59 (60.2)	21 (60.0)	0.880^b^
Lachman preop, n (%)	44 (83.0)	92 (93.9)	33 (94.3)	0.083^c^
Lachman after sedation, n (%)	48 (90.6)	92 (93.9)	33 (94.3)	0.721^c^
Lachman after spinal anestezi , n (%)	53 (100.0)	98 (100.0)	35 (100.0)	>0.05
Pivot Shift preop, n (%)	35 (66.0)	79 (80.6)	29 (82.9)	0.083^c^
Pivot after sedation, n (%)	52 (98.1)	98 (100.0)	35 (100.0)	0.283^c^
Pivot after spinal anestezi , n (%)	53 (100.0)	98 (100.0)	35 (100.0)	>0.05
Lever preop, n (%)	32 (60.4)	79 (80.6)	31 (88.6)	0.003^b^
Lever after sedation, n (%)	33 (62.3)	77 (78.6)	31 (88.6)	0.012^b^
Lever after spinal anestezi , n (%)	40 (75.5)	87 (88.8)	34 (97.1)	0.007^c^
Anterior drawer preop, n (%)	32 (60.4)	67 (68.4)	27 (77.1)	0.253^b^
Anterior drawer after sedation, n (%)	45 (84.9)	88 (89.8)	33 (94.3)	0.356^c^
Anterior drawer after spinal anestezi n (%)	49 (92.5)	94 (95.9)	35 (100.0)	0.122^c^

Spearman’s correlation analysis results showed that all Lever parameters have positive correlation with phase (p<0.01) (Table 4).

**Table 4:** Spearman’s rho correlation analysis between phase, quadriceps atrophy, preop and after sedation Lever and Anterior Drawer parameters **p<0.01

Phase	r	p
Quadriceps atrophy	0.525	0.000**
Lever preop	0.240	0.001**
Lever after sedation	0.216	0.003**
Lever after spinal anesthesia	0.223	0.002**

Wilcoxon signed-rank test results showed that after differences of sedation-after spinal positivity of Pivot and preop-after sedation positivity of Lever were statistically insignificant (p>0.05). All other differences of accuracy of predictive parameters according to preop, after sedation, and after spinal were significant (p<0.05) (Table 5).

**Table 5:** Differences of accuracy of predictive parameters according to preop, after sedation and after spinal anestezi (p values)* *Wilcoxon signed-rank test.

	Preop-after sedation	Preop-after spinal anestezi	After sedation-after spinal anestezi
Lachman	0.046	0.000	0.000
Pivot Shift	0.000	0.000	0.317
Lever	0.739	0.001	0.000
Anterior drawer	0.000	0.000	0.001

## Discussion

In this study, we aimed to evaluate the effectiveness of the diagnostic value of the Lever test on quadriceps atrophy and case phase in patients with ACL injuries, as compared to Lachman, Pivot Shift, and Anterior Drawer tests. In this respect, diagnostic values of Lachman, Pivot Shift, Lever, and Anterior Drawer tests were examined on 189 patients with positive MRI results as the gold standard.

Jarbo et al. [19] reported that the sensitivity ranged from 18-92 for the Anterior Drawer test, 63-93 for the Lachman, and 18-48 for the Pivot Shift test. Deveci et al. [21] reported pre-anesthesia positivity as 94.2% for the Lever test, 80.5% for the Lachman test, 62.3% for the Pivot Shift test, and 60.1% for the Anterior Drawer test. In the same study, they reported 98.4% for the Lever test, 88.7% for the Lachman test, 88.3% for the Pivot Shift test, and 84.2% for the Anterior Drawer test after anesthesia. Mulligan et al. [22] reported sensitivity as 38% and specificity as 72% for Lever Sign in their study. In the same study, sensitivity for the Lachman test was reported as 67% and specificity as 97%. McQuivey et al. [23] reported the sensitivity as 100% for the Lever Sign, while it was 40% for the Lachman test. Lelli et al. [24] reported that all tests for chronic patients have nearly 100% sensitivity, but in acute patients, it is lower in Lachman, Anterior Drawer, and Pivot Shift tests except Lever Sign.

Considering that all cases were positive in our study, the predictive value of the Lever test for preoperative and post-sedation was higher in the quadriceps atrophy positive group (p <0.05). Similarly, the predictive value of Anterior Drawer tests was higher in the group with quadriceps atrophy positive, both preoperatively and after sedation and spinal anesthesia. When compared in general, diagnostic values of Lachman and Pivot shift tests were similar in quadriceps atrophy positive and negative groups, and the differences between them were not statistically significant. However, the presence or absence of quadriceps atrophy had a statistically significant effect on the predictive value of the Lever and Anterior Drawer tests. The quadriceps atrophy affects only one muscle group, but anaesthesia affects all lower limb muscles. Due to this perspective, we think that measurement differences among the distinct tests are obscured after anaesthesia induction. The results of the study showed that the Lever and Anterior Drawer tests were less predictive of predictability in patients without quadriceps atrophy.

In our study, the highest predictive value was measured for the Lachman and Pivot Shift tests after spinal anesthesia. Although the predictive value after spinal anesthesia is the highest in these two methods, it may be stated that the predictive value and preoperative determination of the case are more important. In this respect, the pre-operative predictive value was 97.5% for Lachman test, 87.5% for Pivot Shift test, 95.0% for Lever test and 82.5% for the Anterior Drawer test. Here the Lachman test has the highest predictive value, followed by the Lever, Pivot and Anterior Drawer tests. However, although the second Lever test has a higher predictive value than the Pivot Shift test, it shows a significant difference between those who are quadriceps atrophy positive and those who do not. For this reason, taking into account quadriceps atrophy positivity, it is possible to rank predictive value efficiency from the highest to the lowest as Lachman, Pivot, Lever and Anterior Drawer before the operation. Therefore, quadriceps atrophy positivity affects the diagnostic and predictive values ​​in patients with ACL tears.

In our study, although predictive values of Lachman, Pivot Shift, and Anterior Drawer tests were higher in the chronic group, the differences were not statistically significant. In the Lever parameter alone, the predictive value is higher when the phase is chronic and lower when the phase is acute or subacute. These differences are statistically significant for Lever. Therefore, it can be stated that the predictivity of the Lever test is lower in ACL tears with different phases compared to other methods.

Gürpınar et al. [25] reported a sensitivity value of 80.6% for Lachman, 77.4% for the Anterior Drawer, 51.6% for the pivot, and 91.9 for the lever test in the acute phase. For the chronic phase, these values were reported as 83.9%, 79.0%, 56.5% and 91.9%, respectively. In the chronic phase, the predictive values of all tests are generally higher. In this respect, the research results are similar to our phase study results.

When the tests were compared for different measurement times, the differences in Pivot Shift test after sedation-after spinal anaesthesia and Lever test after preop-sedation were not significant. For all parameters other than these, the predictive value showed a statistically significant increase over time.

Unlike the literature, our study was the first study to investigate the predictive value of Lachman, Pivot Shift, Lever, and Anterior Drawer tests according to acute-subacute-chronic injury phases after ACL injury and quadriceps atrophy cases, as well as being single-centred, small number of cases, meniscus injuries were evaluated in the study. There are also negative aspects such as absence.

## Conclusions

According to the results of this study, the presence or absence of quadriceps atrophy in patients with acute, sub-acute, or chronic ACL injury has a significant effect on the predictive value of the Lever test. The predictive value of the Lever test is higher than the other tests in patients with quadriceps atrophy. This indicates that there may be other conditions that may affect other predictive parameters in patients with ACL rupture. For this reason, it is clear that univariate analyses may give incorrect results in studies conducted to demonstrate predictive value, and multivariate analyses would be more accurate.
